# Hemodynamic Responses to Resistance Exercise with Blood Flow Restriction Using a Practical Method Versus a Traditional Cuff-Inflation System

**DOI:** 10.3390/ijerph191811548

**Published:** 2022-09-14

**Authors:** Lee J. Winchester, Morgan T. Blake, Abby R. Fleming, Elroy J. Aguiar, Michael V. Fedewa, Michael R. Esco, Ryan L. Earley

**Affiliations:** 1 Exercise Physiology Laboratory, Department of Kinesiology, The University of Alabama, Tuscaloosa, AL 35487, USA; 2 Department of Biology, The University of Alabama, Tuscaloosa, AL 35487, USA

**Keywords:** occlusion, BFR, tissue flossing, hypoxia, resistance exercise

## Abstract

The aim of this study was to examine the potential differences in acute hemodynamic responses and muscular performance outcomes following resistance exercise between traditional blood flow restriction (TRA_BFR_) and a novel band tissue flossing method (BTF_BFR_). METHODS: Fifteen healthy young adults (23.27 ± 2.69 years) visited the lab for three sessions (≥72 h apart). Each session’s exercise consisted of three sets of 20 maximum-effort seated leg extensions and flexions with one of three conditions: control (CON), TRA_BFR_ (50% limb occlusion pressure (LOP)), or BTF_BFR_. During TRA_BFR_ and BTF_BFR_ sessions, occlusion was applied immediately prior to exercise and removed immediately after. Heart rate was collected prior to exercise, after onset of occlusion, immediately after exercise, and one-minute after removal of occlusion. Ultrasonography was performed prior to, and at least 30 s after, occlusion. RESULTS: BTF_BFR_ caused greater reductions in arterial distance (14.28%, *p* = 0.010) and arterial area (28.43%, *p* = 0.020) than TRA_BFR_. BTF_BFR_ was able to significantly reduce arterial flow below pre-occlusion values, while TRA_BFR_ did not. Both conditions caused significant elevations in heart rate following occlusion (TRA_BFR_: +4.67 bpm, *p* = 0.046 and BTF_BFR_: +6.07 bpm, *p* = 0.034), immediately post-exercise (TRA_BFR_: +56.93 bpm, *p* < 0.001 and BTF_BFR_: +52.79 bpm, *p* < 0.001) and one-minute post-exercise (TRA_BFR_: +15.71, *p* = 0.003 and BTF_BFR_: +14.57, *p* < 0.001). Only BTF_BFR_ caused significant reductions in performance as measured by average power per repetition. CONCLUSIONS: BTF_BFR_ causes a more exaggerated decrease in arterial blood flow as well as muscular power when compared to traditional TRA_BFR_ at 50% of LOP.

## 1. Introduction

Blood flow restriction (BFR) devices have been popularized in recent years following research highlighting gains in muscular strength and hypertrophy using resistance loads as low as 20% of one repetition maximum (1 RM) that are comparable to non-BFR resistance loads ≥ 65% of 1 RM [1]. Blood flow restriction devices fully occlude venous return of metabolites while partially preventing the inflow of oxygenated arterial blood [2] through inflation of a pneumatic cuff that is applied proximally to the limb. Proper BFR application during exercise seems to enhance exercise pressor response, resulting in elevated heart rate [3] and blood pressure [4], but a reduction in arterial blood flow distal to the site of occlusion [5], despite the alterations to central hemodynamics.

The blood flow reduction due to BFR application results in acute hypoxic conditions that increase metabolic stress, recruitment of fast-twitch muscle fibers, production of reactive oxygen species, and production of anabolic hormones [6]. Enhancement of these physiological responses to resistance exercise is known to enhance muscle protein synthesis and improve muscle strength and hypertrophy [6]. Furthermore, an acute decrease in muscular performance is commonly observed during resistance exercise with BFR [7,8,9,10], which is likely due to metabolite accumulation rather than muscular damage [9,11], though the effects of BFR on muscular damage is heavily debated as results are inconsistent [12,13]. This explains why BFR is effective at increasing muscular hypertrophy at low-resistance loads, since intensities <65% of 1 RM are not typically associated with muscle hypertrophy [1].

Traditionally, BFR is implemented through the inflation of a pneumatic cuff system to occlude blood flow to the distal musculature. However, these devices typically cost thousands of dollars and are not feasible for use within practical settings. A newer, less studied method of BFR, called band tissue flossing (BTF_BFR_), has emerged. This process involves the use of elastic bands that are commonly applied to enhance joint function and mobility [14]. For example, the application of elastic bands to the ankle results in small-to-moderate improvements in range of motion during dorsiflexion (−7°), and plantarflexion (+5°), as well as vertical jump height (+0.04 m) [15].

For inducing BFR, the elastic bands are wrapped around the proximal portion of an upper limb to occlude and prevent distal blood flow [16,17,18]. The technique seems to serve as an affordable alternative to traditional cuff-inflation systems and, hence, a practical approach for BFR exercise. However, the research regarding the effect of BTF_BFR_ on hemodynamic responses and muscular performance during exercise is incomplete. Among the limited investigations, Loenneke et al. utilized elastic bands as a means of inducing venous occlusion during knee extension resistance exercise and found no elevations in blood lactate compared to control, yet the exercise intensity was described as “low” [16]. Furthermore, the elastic wraps were only applied to the thighs during the sets and removed during the rest periods [16]. This contrasts with the consensus of leaving the occlusion devices in place during and in between the exercise sets. In addition, although the study provided photographs of the application process and described the needed pressure as “low” [17], no written description of tourniquet application procedure was provided, making interpretation difficult. Moreover, other researchers that have utilized elastic resistance bands have implemented a protocol of applying tension based on perceived tightness (i.e., six or seven out of a scale of one to 10) [19], but this has been shown to provide unreliable and inconsistent estimates of occlusion [20].

Consequentially, a standardized BTF_BFR_ application protocol has yet to be developed. Therefore, quantifying the reduction in blood flow in these scenarios, replicating the observed results from previous research, and employing a practical method with confidence is difficult. However, it is well understood that standardized protocols emerge following numerous investigations on a given topic. Thus, as with any novel method, further research is needed to advance this area of study. As such, the purpose of this study was to observe potential differences in the acute hemodynamic responses and muscular performance outcomes following resistance exercise when implementing a proposed, simplified method using elastic bands compared to a traditional automated BFR device (TRA_BFR_). The results will assist previous foundational research, which, collectively, will eventually lead toward a standardized, practical approach for BFR exercise. It was hypothesized that application of the elastic bands will induce similar physiological responses at rest and during resistance exercise compared to a traditional cuff inflation system at 50% of Limb Occlusion Pressure (LOP).

## 2. Materials and Methods

### 2.1. Study Design

The aim of this study was to utilize a practical BTF_BFR_ application protocol to examine the potential differences in acute hemodynamic responses and muscular performance outcomes following resistance exercise between TRA_BFR_ and BTF_BFR_. The study utilized a randomized, crossover, repeated measures design. The measurements chosen for this study incorporated several measures of arterial flow and cardiovascular response by ultrasonography and heart rate monitoring, as well as muscular fatigue by isokinetic dynamometry. The conditions were assigned in randomized order to each participant to ensure the results observed were not due to a training effect.

### 2.2. Subjects

The protocol utilized in this study was approved by the University of Alabama Institutional Review Board. Participant written consent was obtained prior to the first data collection session. All participants were informed of the benefits and risks of the investigation prior to signing the informed consent. A power analysis (G*Power v.3.1.9.6, Heinrich Heine University Düsseldorf, Düsseldorf, Germany) revealed a need for 15 participants to obtain a large effect size (f = 0.4, α = 0.05, β = 0.9) based on pilot data from our first three participants. Fifteen young adults participated in this study (see Table 1 for descriptive statistics). Participants were non-smokers that did not require medical clearance to engage in exercise according to the ACSM pre-participation screening tool [21]. Pregnant women, individuals with symptoms or diagnoses of cardiovascular disease, and individuals with metabolic disease were excluded from this study. No recruited participants met the exclusionary criteria.

### 2.3. Procedures

Each participant engaged in three different data collections over a two to three week period, with each session being no less than 72 h apart to ensure adequate recovery time, though not exceeding two weeks between sessions. Each of the sessions involved exercise under only one of the three conditions: control (CON), BTF_BFR_, and TRA_BFR_. The order for the occluding applications was randomized for each participant using Microsoft Excel v.16.48.

During the first session only, anthropometric measurements including height (SECA 213, Seca Ltd., Hamburg, Germany) and weight (Tanita BWB-800, Tanita Corporation, Tokyo, Japan) were measured once to the nearest 0.1 cm and kg, respectively. For descriptive purposes, body mass index (BMI) was calculated as kg/m^2^, with participants categorized as normal weight (18.50–24.99 kg/m^2^), overweight (25.00–29.99 kg/m^2^), and obese (≥30.00 kg/m^2^). Relative adiposity (%Fat) was determined using a bioimpedance spectroscopy device (ImpediMed SFB7, Carlsbad, CA, USA) [22]. Thigh length and circumference were measured using a flexible, tension-sensitive, non-elastic vinyl tape measure (Gulick, Lafayette Instrument Co, Lafayette, IN, USA) to the nearest 0.1 cm. Thigh length was measured from the top of the patella to the inguinal crease, whereas thigh circumference was measured at 2/3 thigh length in the proximal direction.

For all three sessions, participants were outfitted with a heart rate (HR) monitor (Polar H10; Polar Electro, Kempele, Finland) and HR was recorded for the entirety of each data collection session. After being outfitted, the participant was seated and completed a 24-h recall form detailing recent consumption of foods, drinks, and dietary supplements, as well as physical activity and sleep duration. Participants remained seated, and resting HR was recorded after 5 min of rest (Pre-). Next, the participant was seated upright on the isokinetic dynamometer (Humac Norm, CSMi, Stougton, MA, USA) in the manufacturers recommended orientation for knee extension and flexion. B-mode ultrasound imaging (Philips iU22 Ultrasound, Bothell, WA, USA) was performed to determine a baseline (Pre-) lumen distance (Dist; reported in cm), area (reported in cm^2^), volume (Vol Flow; reported in cc/min), and time-averaged mean velocity (TAMV; reported in cm/s) of the tibial artery, adjacent to the medial malleolus, on the participant’s dominant (involved) leg. After determining the baseline ultrasound measurement, the TRA_BFR_ cuff was inflated or BTF_BFR_ wrapped on the thigh to cause occlusion. After at least 30 s of occlusion, the ultrasound measurements were re-evaluated (Post-). This was only performed during the TRA_BFR_ and BTF_BFR_ conditions, with the baseline measurements serving as the control comparison for the post-TRA_BFR_ or BTF_BFR_ assessment. Furthermore, in the TRA_BFR_ and BTF_BFR_ conditions only, a second pre-exercise heart rate measurement was recorded at the same time as the ‘post-occlusion’ ultrasound measurements.

The exercise protocol common to all three data collection sessions involved three sets of 20 maximal knee extensions/flexions at 90° per second on the isokinetic dynamometer with their dominant leg, with 30 s of rest between sets. During exercise, the isokinetic dynamometer recorded average power per repetition for all sixty repetitions within a session. This protocol was chosen to maximize the rate of fatigue that could be observed through the power data on the Humac dynamometer. After completion of the third set, an immediate post-exercise (ImmPost) heart rate measurement was recorded, followed by removal of occlusion. One minute after the occlusion device had been removed (or 1-min post-exercise in the control condition), a final heart rate measurement was recorded (1-Min Post).

### 2.4. TRA_BFR_ Application

The TRA_BFR_ device used in this study was the FDA-approved Delfi PTS II (Owens Recovery Science, San Antonio, TX, USA). The Delfi PTS II system is an automated cuff system that accurately determines LOP [23], the pressure required to completely occlude arterial blood flow in the lower limb, while at rest. The Delfi cuff was applied to the proximal thigh of the participant’s dominant limb to determine LOP. Once LOP was known, the cuff system was inflated to 50% of the participant’s personal LOP. Although 80% LOP is commonly used in the lower limbs according to the manufacturer, 50% LOP was chosen as the comparison pressure with the Delfi PTS II system as a precaution to ensure participant safety, since participants were asked to perform the exercise at maximal effort for each set, as opposed to the low-intensity resistance exercise traditionally associated with TRA_BFR_ training. The cuff remained inflated for the duration of all three sets of exercise and deflated after completion of the third set.

### 2.5. BTF_BFR_ Application

**Figure 1:**
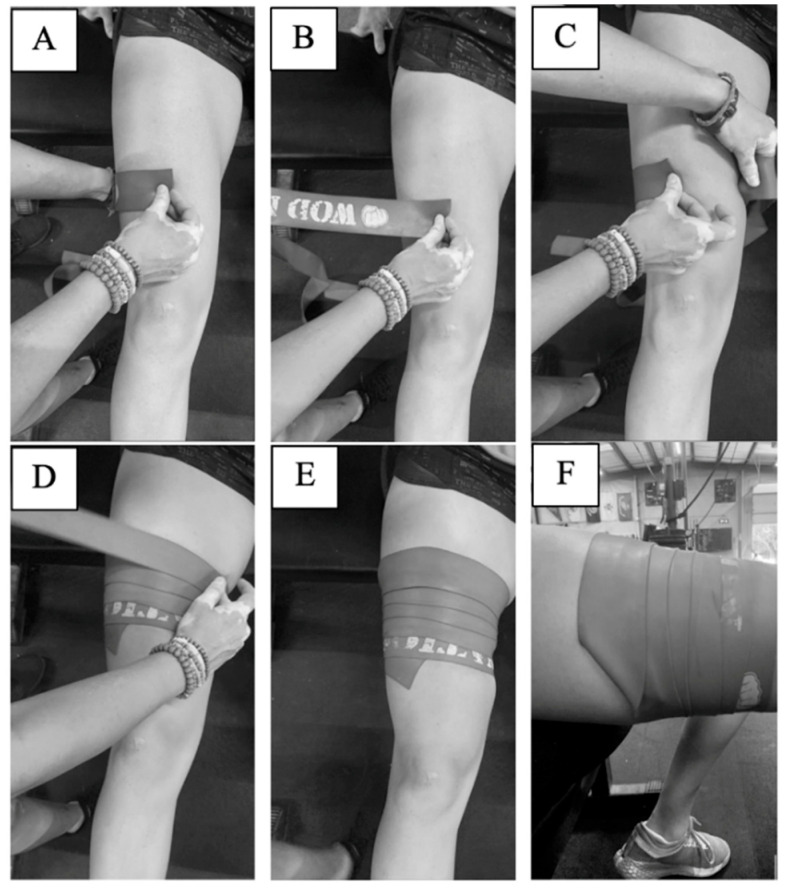
BTF_BFR_ wrapping protocol. (**A**): Starting 1/3 of the thigh length from the top of the patella, measure the band to ½ the participant’s thigh circumference. (**B**): Marking that distance with the thumb, lift the band off the participant’s skin and stretch the band. (**C**): Stretch that distance of the band around ¾ of the participant’s thigh circumference and apply pressure to keep in place. (**D**,**E**): Continue this process, with each layer overlapping the previous by ½ the width of the band. (**F**): Tuck the loose end under the layer prior.

This study used heavy strength WOD Nation Compression Muscle Floss (seven-feet long, two-inches wide) (Suring, WI, USA). A practical protocol was developed and implemented to achieve the “50% wrap, 50% overlap” methodology commonly described on the websites of many band manufacturers, in order to achieve consistent application between participants. Specifically, wrapping occurs in the distal-to-proximal direction, stretching the bands an additional 50% of its original length and overlapping the segments below it by 50% of its width. The procedure designed and utilized in the present study is illustrated in Figure 1 for visual reference and described in detail below.

First, the length of the participant’s thigh was measured from the top of the patella to the inguinal crease. This length was then divided into thirds. At a distance 1/3 of the length of the thigh from the top of the patella, a horizontal line was drawn with washable marker to mark where wrapping of the BTF_BFR_ band would begin. This application location was chosen to ensure participant comfort with the application procedure and to keep the protocol as practical and accessible as possible for individuals wishing to use this technique while engaging in their daily fitness routine, which may occur at a public facility. At a distance 2/3 the length of the thigh from the top of the patella, thigh circumference was measured. The circumference measurement (x) was then divided into quarters, and vertical lines were drawn down the participant’s leg delineating these quarter-markings. While seated, but with the thigh not touching the chair, the band was then placed atop the participant’s leg at the first quarter mark. The band was then wrapped unstretched around two quarters of the participant’s thigh, and the researcher’s thumb was placed at that position on the band, marking 0.5x circumference (A). The band was then lifted slightly off the participant’s skin (to prevent skin abrasion) (B), and that length of the band (0.5x) was stretched around 0.75x of the participant’s thigh (C). This procedure was repeated for the entire length of the band. The overlap the band had with the piece of band underneath was 0.5y, where “y” is the width of the band (D–E). When the band’s end was reached, the loose end was tucked underneath the layer before it to prevent unraveling (F). In all, this method increases the length of the elastic band to approximately 150% of its resting length. This procedure provides a repeatable band application that can be utilized by fitness enthusiasts and for future research. To help maintain consistency in the tension applied between individuals, each participant used a new, unused band, and the bands were applied by the same researcher for every participant. For additional precaution, no occluding application was in place for more than 8 min at a time.

### 2.6. Control Condition

For the control condition (CON), no occlusive device was applied to the limb prior to or during exercise. During this session, all procedures were the same as the occlusion device sessions, but without the pre-post occlusion ultrasound measurements and post-occlusion heart rate.

### 2.7. Statistical Analyses

All statistical analyses were performed using SPSS for Windows v.27 (IBM Corporation, Somers, NY, USA). Mean (M) and standard deviation (SD) values were generated for all outcome measures. Paired samples t-tests were used to test for differences in post-occlusion, pre-exercise changes between the occlusion conditions, including arterial flow and heart rate. A standardized mean effect size (ES) calculated as (M_BTF_ − M_TRA_/SD_BTF_) for between-group comparisons, whereas within-conditions were calculated as (M_Pos*t*_ − M_Pre_/SD_Pre_), and qualitatively described as small, moderate, and large (ES = 0.2, 0.5, and 0.8, respectively) [24]. Normality for these variables was determined using the Shapiro Wilk test and visual inspection of residual histograms and QQ-plots. When the normality assumption was violated (‡), the Kruskal-Wallis test was used.

Repeated measures ANOVAs were used for the other continuous variables to compare raw values and change scores of measures between- and within- conditions. Sphericity was tested using Mauchly’s *W*. If sphericity was violated, the non-parametric Kruskal-Wallis independent samples test was used. If a main effect was found, a post-hoc analysis with Bonferroni correction was utilized to determine between-condition differences. Statistical significance was indicated using an α level of *p* < 0.05 and was used for all tests.

## 3. Results

### 3.1. Arterial Flow

**Figure 2:**
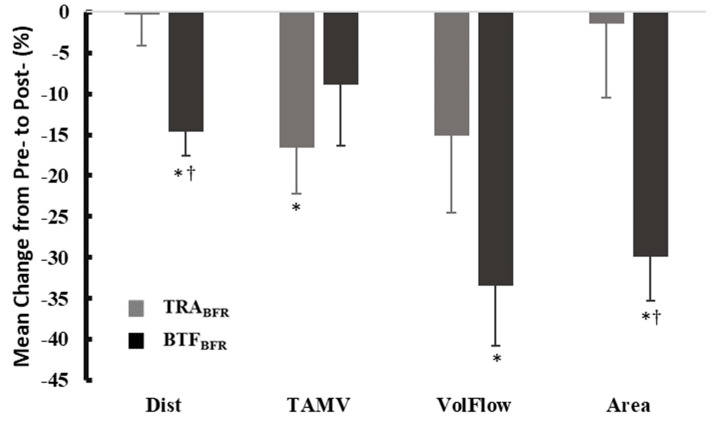
Mean percent change in hemodynamic responses between conditions (M ± SEM). † = Significantly different from TRA_BFR_; * = Significant difference in pre-/post-measures within condition. TAMV = Time-averaged mean velocity; VolFlow = volume flow; CON = control; TRA_BFR_ = traditional blood flow restriction; BTF_BFR_ = band tissue flossing. Statistical significance is noted at an alpha level of *p* < 0.05.

The BTF_BFR_ condition, but not the TRA_BFR_ condition, caused a significant decrease (*p* = 0.001) in volume flow from the baseline (Pre) value, with no differences between conditions (ES = −0.721 vs. −0.335, *p =* 0.092). The BTF_BFR_ condition caused a greater decrease in arterial distance than the TRA_BFR_ condition (ES *=* −0.849 vs. −0.060, respectively, *p =* 0.007). In contrast, a small-moderate decrease in TAMV was observed in the TRA_BFR_ condition (*p* = 0.029), but not the BTF_BFR_ condition, with no differences between the conditions (ES *=* −0.212 vs. −0.325, *p = 0*.662). BTF_BFR_, but not TRA_BFR_, resulted in a decrease in arterial area Pre- vs Post (*p* =< 0.001), with the BTF_BFR_ change being significantly different from TRA_BFR_ (ES = −0.884 vs. −0.099, *p =* 0.007). Changes in arterial flow are displayed in Table 2 and Figure 2.

### 3.2. Heart Rate

**Figure 3:**
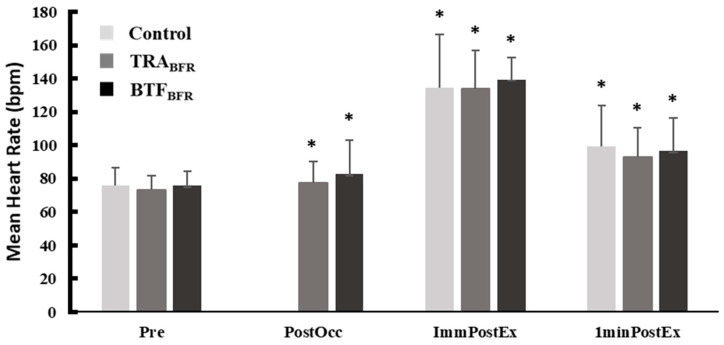
Heart rate in each condition across different time points (M ± SD). No post-occlusion HR data exists for the CON condition, as no occlusion was applied. * = Significant from pre-measurement. TRA_BFR_ = traditional blood flow restriction; BTF_BFR_ = band tissue flossing. Statistical significance is noted at an alpha level of *p* < 0.05.

HR was significantly increased for BTF_BFR_ (*p* = 0.034) and TRA_BFR_ (*p* = 0.046) from Pre- to Post- occlusion, within conditions. HR was also significantly increased from Pre- values, within condition, for CON, TRA_BFR_, and BTF_BFR_ at ImmPost and 1-Min Post time points. There were no significant differences among resting HR data (Pre-) or post-occlusion HR data (Post-) between conditions. In addition, the change in HR was not different between conditions Post-, ImmPost, or 1-Min Post (all *p* > 0.05). Despite the moderate ES, no statistically significant change in HR was observed in the BTF_BFR_ or TRA_BFR_ conditions Post (ES = 0.492 and 0.668, respectively; both *p* > 0.05). A large increase in HR was observed ImmPost and 1-Min Post under all conditions (Table 3). Between-condition ANOVA revealed no significant differences among raw values ImmPost- nor 1-Min Post-exercise between conditions. Furthermore, the change in HR data is summarized in Figure 3.

### 3.3. Average Power Per Repetition

There were no significant differences in average extension power per repetition between sets during both the CON and TRA_BFR_ conditions. However, a main effect was found during the BTF_BFR_ condition in which average power per repetition was significantly less in sets 2 and 3 than during set 1, (*p* = 0.035 and *p* < 0.001), respectively. In addition, there were no significant differences in average power per repetition during flexion within any of the conditions.

**Figure 4:**
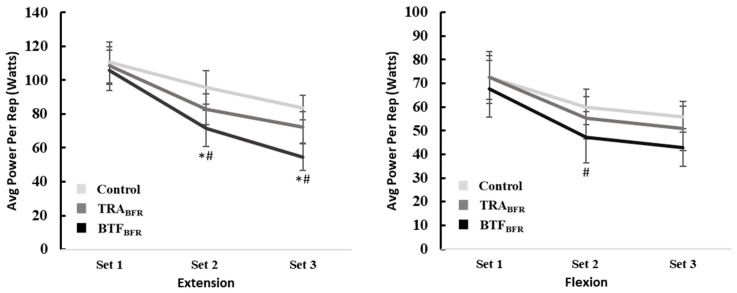
Average power per rep during extension and flexion (M ± SEM). # = Significantly different from CON; * = Significant difference within condition. CON = control; TRA_BFR_ = blood flow restriction; BTF_BFR_ = band tissue flossing. Statistical significance is noted at an alpha level of *p* < 0.05.

When examining the average extension power per repetition between groups, there were no significant differences between the groups during set 1. In set 2, mean average power per repetition during BTF_BFR_ was significantly less than during CON (*p* = 0.004). Similarly, mean average power per repetition during set 3 was significantly less in BTF_BFR_ compared to CON (*p* < 0.001). There were no significant differences between BTF_BFR_ and TRA_BFR_ or TRA_BFR_ and CON during sets 2 and 3. When examining the average flexion power per repetition between groups, there were no significant differences between conditions during sets 1 and 3. In set 2, mean average power per repetition was significantly less in BTF_BFR_ compared to CON (*p* = 0.009). There were no significant differences between BTF_BFR_ and TRA_BFR_ or TRA_BFR_ and CON during set 2. Mean values for each set by condition are shown in Table 4. The responses across each set are visually displayed in Figure 4.

## 4. Discussion

The primary finding from this current study was that BTF_BFR_, consistently stretched to 150% of resting length throughout the wrapping protocol, causes a similar or slight decrease in arterial volume flow and arterial size, as well as accelerated decrements in muscular power, when compared to TRA_BFR_ at 50% of LOP and the control condition. Therefore, the BTF_BFR_ technique, as described in this study, is a practical approach for blood flow restriction that can be easily implemented into daily fitness routines.

There is very limited data available regarding the use of elastic bands (BTF_BFR_) for occlusion of blood flow during exercise training and few standardized means of application. For instance, Loenneke et al. demonstrated that blood flow restriction with similar elastic bands did not induce metabolic stress during knee flexion resistance exercises [16] or walking [18] when compared to the control conditions without occlusion. However, there are a few methodological differences between the present and the previous studies. First, both exercise conditions were described as having a “low” exercise intensity [16,18], while the isokinetic exercise trials of the current study were performed at maximal effort. Second, the elastic band was removed in between the sets of knee extension in Loenneke et al. [16], yet in the current study they remained in position during the entire duration (i.e., exercise sets and rest periods) of the BTF_BFR_ experimental trials on the isokinetic device. Third, and likely of most importance, the present study included the TRA_BFR_ condition for comparison to a criterion. The two former studies mentioned herein did not include a traditional blood flow restriction device. Furthermore, it should be noted that the use of band tissue flossing is a newer and more practical approach for inducing blood flow restriction, which explains the limited research. Because of the novelty, standardized procedures for its application has yet to emerge. Previous attempts toward standardization have employed a tension application of the elastic band by a perceived tightness scale of six to seven out of 10 [19]. However, more recent research has suggested this approach as being unreliable due to producing inconsistent estimates of occlusion [20] and hence, potentially too subjective. Thus, the wrapping technique chosen in this study that resulted in a 150% increase in band length was an attempt toward a more objective approach than previous work, while also keeping the focus on practicality. Nonetheless, additional research is needed to produce appropriate standardized procedures for band tissue flossing that can be competently incorporated across the masses.

In the current study, as expected, HR was significantly increased after inflation of the TRA_BFR_ cuffs or application of BTF_BFR_. This is in agreement with past literature emphasizing the enhanced pressor response with TRA_BFR_ application [3,4], In contrast, no significant differences in HR existed between conditions at ImmPost and 1-Min Post time points. Although plentiful research demonstrates elevated HR response with TRA_BFR_ application, Bazgir et al. found that four sets of eccentric resistance exercise with TRA_BFR_ resulted in no significant increase HR in comparison to the control condition [4], which is in agreement with the findings of this current study.

TRA_BFR_ at 50% was not significantly different from Pre- (pre-occlusion) values regarding changes in arterial volume flow; conversely, BTF_BFR_ was able to cause significant reductions in flow compared to Pre- occlusion values. While TRA_BFR_ at 50% LOP caused a 12.36% reduction in volume flow at the tibial artery compared to Pre-, BTF_BFR_ caused a 32.51% reduction. Moreover, BTF_BFR_ caused a reduction in arterial area and distance that was significantly lower than TRA_BFR_, further emphasizing the ability of BTF_BFR_, using this standardizable protocol, to effectively reduce arterial flow. Previous research has demonstrated that relatively wide (10–12 cm) TRA_BFR_ cuffs tend to occlude flow at lower pressures than thinner cuffs (5 cm), likely due to the larger contact area with the underlying blood vessels [5]. In the current study, although the TRA_BFR_ condition utilized an 11.4 cm wide cuff, which is wide by traditional TRA_BFR_ standards, the BTF_BFR_ application resulted in an occlusion area that compressed a comparatively larger portion of the thigh. This may also explain, at least in part, why BTF_BFR_ was effective at significantly reducing flow and diameter in the distal vasculature. A recent study by Vogrin et al. identified “high pressure” using a BTF_BFR_ band as 150–210 mmHg, depending on thigh circumference [25]. This “high pressure” was assumed to be comparable to TRA_BFR_ occlusion at 60%, (though blood flow was not measured in the Vogrin study). Based on the Vogrin study and BTF_BFR_’s demonstration of occluding blood flow more effectively than TRA_BFR_ at 50% LOP in the present study, the use of BTF_BFR_ in this protocol is likely applying “high pressure”, though exact pressure was unable to be measured in the present study.

The high level of occlusion by BTF_BFR_ is further demonstrated in this study by an increased rate of muscular fatigue. It was found that BTF_BFR_ was more fatiguing than CON, with significantly lower average power per rep. Interestingly, TRA_BFR_ was not significantly different from either BTF_BFR_ or CON. This is in agreement with previous literature that demonstrates an increased rate of fatigue with TRA_BFR_ application, even when occlusion is applied intermittently during the sets [9]. Conversely, other literature has cited an increase in power with the application of occlusion [26]; however, this discrepancy is most likely due to differences between protocols. In a study by Gepfert et al., TRA_BFR_ was removed for 3 min between each set in a 3 × 3 back squat protocol, allowing time for reperfusion and probable clearance of local metabolic byproducts that was not allowed in the current protocol [26]. Furthermore, their study utilized 150% of arterial occlusion pressure when obtaining these results, while 100% arterial occlusion pressure results in no difference from control, which is much closer to pressures used in the current study.

The exercise pressor reflex (EPR) may also influence response to occlusion. During intense resistance exercise, blood vessels within the active muscle vasodilate via active hyperemia to accommodate the increased need for oxygen delivery [27]. Concurrently, sympathetic innervation promotes vasoconstriction of vessels peripheral to the active muscle in an effort to shut blood flow to areas of priority. BFR reduces outflow of this blood in order to accentuate local metabolic stress, a mechanism hypothesized to stimulate the upregulation of hypertrophic pathways [6]. The EPR is known to be engaged even at mild-intensity exercise and presumed to be exaggerated under BFR conditions [28]. EPR is known to be influenced by a variety of factors, including mental stress [29], dietary sodium intake [30], blood pressure [31,32], muscle mass and contraction intensity [33], and training status [34], giving this the potential to be both extremely variable and influential in creating differences between participants. Therefore, caution must be used when implementing any sort of BFR protocol with populations at risk of cardiovascular disease.

Although this study attempted to control for as many variables as possible, it is not without design limitations. One of the primary limitations of this study is that we were unable to measure the amount of pressure provided by the BTF_BFR_ bands and, therefore, specific pressure application could have varied between participants. Since this protocol is meant to serve as a practical application that people could easily utilize in a fitness facility, the exact tension of the applied BTF_BFR_ bands was not measured. However, to provide a repeatable application across all participants, the band was consistently lengthened to 150% of its resting length throughout the application procedure. The typical fitness enthusiast is unlikely to have readily available access to any device that could measure band tension and, therefore, we chose to replicate BTF_BFR_ utility in a manner that could be easily utilized by this population. Another limitation is that the response to occlusion may be individualized based on a variety of factors, including body composition and response of the vasculature. Vogrin et al. divided participants into three groups based on thigh circumference (51–55.9 cm, 56–59.9 cm, and >60 cm) to assess for differences in response to occlusion between groups [25]. This was not feasible in the present study due the relatively small sample size and limited range of thigh circumference values. Additionally, for this study, an isokinetic dynamometer was used as the exercise modality rather than a more practical, traditional resistance exercise. However, the dynamometer was needed to obtain set-to-set power data throughout the study protocol, which is critical for determining efficacy of the BTF_BFR_ application. Finally, most of the participants used in this study were healthy and of college-age. Because of this, the results of this study may not be generalizable to children and adults outside of the included age range, nor to adults with different health conditions.

## 5. Conclusions

Results from this present study demonstrate that BTF_BFR_ decreases blood flow, arterial diameter, and muscular power during resistance exercise to a similar or greater extent than TRA_BFR_ set to 50% of participant LOP. This study proposes a practical protocol for BTF_BFR_ wrapping that could be implemented in future studies and could be easily implemented by fitness enthusiasts. The results of this study suggest that BTF_BFR_ could serve as a low-cost, effective alternative for individuals wishing to train with blood flow occlusion outside of a clinical or laboratory setting. However, there is still much research to be done pertaining to the physiological responses to BTF_BFR_ application. Future use of this BTF_BFR_ protocol will seek to build the body of literature regarding its efficacy for serving as a practical, affordable alternative to traditional pneumatic cuff systems so that it can develop into a standardized protocol. Research implementing this protocol with more traditional exercise routines and training studies are already in development. Evaluation of tolerability measures, such as perceived discomfort, rating of perceived exertion, and a 10-point pain scale should be utilized in future studies to increase the body of knowledge surrounding the efficacy of this BTF_BFR_ method.

## Figures and Tables

**Table 1:** Participant characteristics (M ± SD).

Characteristic	Total Sample	Female	Male
N	15	6	9
Age (y)	23.27 ± 2.69	23.67 ± 2.88	23.00 ± 2.69
Weight (kg)	81.70 ± 17.40	68.00 ± 5.70	92.00 ± 14.90
Height (cm)	177.30 ± 9.20	169.4 ± 6.00	182.50 ± 6.90
BMI (kg/m^2^)	25.72 ± 3.62	23.71 ± 1.99	27.56 ± 3.66
% Fat	26.27 ± 6.37	28.75 ± 7.59	24.61 ± 5.23
Thigh Length (cm)	42.07 ± 4.23	38.17 ± 1.72	44.67 ± 3.24
Thigh Circumference (cm)	59.93 ± 6.04	56.67 ± 2.64	62.11 ± 6.79

**Table 2:** Changes in arterial flow following blood flow restriction and band tissue flossing (M ± SD).

	TRA_BFR_		Within-Condition *p* Value	BTF_BFR_		Within-Condition *p* Value	Between-Condition *p* Value
	Pre	Post		Pre	Post		
Dist (cm)	0.245 ± 0.039	0.243 ± 0.042	0.804	0.263 ± 0.050	0.221 ± 0.030	<0.001 *	0.007 *
TAMV(cm/s)	5.475 ± 3.186	4.441 ± 2.658	0.029 *	6.054 ± 3.457	5.321 ± 2.489	0.256	0.662
Vol Flow (cc/min)	15.366 ± 9.520	12.179 ± 7.162	0.135	20.549 ± 10.49	12.985 ± 7.633	0.001 *	0.092
Area (cm^2^)	0.048 ± 0.016	0.047 ± 0.019	0.711	0.056 ± 0.020	0.038 ± 0.013	<0.001 *	0.007 *

TRA_BFR_ = traditional blood flow restriction; BTF_BFR_ = band tissue flossing; Pre = pre-occlusion; Post = post-occlusion; Dist = lumen distance; TAMV = time-averaged mean velocity; Vol Flow = volume flow; Area = lumen area; * denotes statistical significance. Statistical significance is noted at an alpha level of *p* < 0.05.

**Table 3:** Changes in heart rate following occlusion and exercise (M ± SD).

			Pre vs. Post *p*-Value		ImmPost vs. Pre *p*-Value		1-Min Post vs. Pre *p*-Value
**CON**	Pre	Post		ImmPost		1-Min Post	
HR (bpm)	76.1 ± 10.6	-	-	134.2 ± 32.1	<0.001 *	99.2 ± 24.7	<0.001 *
**TRA_BFR_**	Pre	Post		ImmPost		1-Min Post	
HR (bpm)	73.0 ± 8.9	77.7 ± 12.7	0.046 *	134.0 ± 22.9	<0.001 *	92.8 ± 17.8	0.005 *
**BTF_BFR_**	Pre	Post		ImmPost		1-Min Post	
HR (bpm)	76.0 ± 13.9	82.8 ± 12.0	0.034 *	139.5 ± 30.9	<0.001 *	96.6 ± 22.6	0.001 *

CON = control; TRA_BFR_ = traditional blood flow restriction; BTF_BFR_ = band tissue flossing; HR = heart rate; bpm = beats per minute; Pre = pre-occlusion; Post = post-occlusion; ImmPost = immediately post-exercise; 1-Min Post = 1-min post-exercise; * indicates statistical significance. Statistical significance is noted at an alpha level of *p* < 0.05.

**Table 4:** Changes in average power per repetition during each of the three exercise sets (M ± SD).

				Within-Condition Main Effect *p*-Value
**CON**	Set 1	Set 2	Set 3	
Avg Power Per Rep, Extension (W)	110.5 ± 45.6	95.8 ± 37.4	83.9 ± 27.6	0.259
Avg Power Per Rep, Flexion (W)	72.5 ± 34.7	60.0 ± 27.7	55.9 ± 23.8	0.343
**TRA_BFR_**	Set 1	Set 2	Set 3	
Avg Power Per Rep, Extension (W)	108.6 ± 41.2	82.9 ± 33.5	72.2 ± 34.8	0.068
Avg Power Per Rep, Flexion (W)	72.5 ± 31.8	55.3 ± 24.3	50.9 ± 22.5	0.085
**BTF_BFR_**	Set 1	Set 2	Set 3	
Avg Power Per Rep, Extension (W)	105.7 ± 44.7	71.5 ± 40.7 *#	54.5 ± 29.5 *#	0.004 *
Avg Power Per Rep, Flexion (W)	67.8 ± 31.9	47.2 ± 23.1 #	42.8 ± 19.8	0.070

CON = control; TRA_BFR_ = traditional blood flow restriction; BTF_BFR_ = band tissue flossing; Avg = average; Rep = repetition; W = watts; * denotes significance between sets. **#** denotes significant difference from CON. Statistical significance is noted at an alpha level of *p* < 0.05.

## Data Availability

The data presented in this study are available upon request from the corresponding author.
